# 2-(5-Chloro-2-oxoindolin-3-yl­idene)hydrazinecarbo­thio­amide

**DOI:** 10.1107/S1600536813033369

**Published:** 2013-12-14

**Authors:** Viviane Conceição Duarte de Bittencourt, Juliano Rosa de Menezes Vicenti, Jecika Maciel Velasques, Priscilla Jussiane Zambiazi, Vanessa Carratu Gervini

**Affiliations:** a Escola de Quimica e Alimentos, Universidade Federal do Rio Grande, Av. Italia, km 08, Campus Carreiros, 96203-900 Rio Grande-RS, Brazil; b Departamento de Quimica, Universidade Federal de Santa Maria, Av. Roraima, Campus, 97105-900 Santa Maria-RS, Brazil

## Abstract

The title mol­ecule, C_9_H_7_ClN_4_OS, is almost planar, with an r.m.s. deviation of 0.034 (2) Å for the mean plane through all the non-H atoms. Intra­molecular N—H⋯O and N—H⋯N hydrogen bonds form *S*(6) and *S*(5) ring motifs, respectively. In the crystal, mol­ecules are assembled into inversion dimers through pairs of co-operative N—H⋯Cl inter­actions. These dimers are connected along the *b* axis by N—H⋯O and N—H⋯S hydrogen bonds, generating layers parallel to (103). The layers are further connected along the *a* axis into a three-dimensional network, through weak π–π stacking inter­actions [centroid–centroid distance = 3.849 (2) Å].

## Related literature   

For the synthesis of the title compound, see: Qasem Ali *et al.* (2011 ▶). For similar hydrazinecarbo­thio­amide crystal structures, see: Bandeira *et al.* (2013 ▶); Ali *et al.* (2012 ▶); de Oliveira *et al.* (2012 ▶). For the biological activity of isatin and derivatives, see: Cerchiaro & Ferreira (2006 ▶).
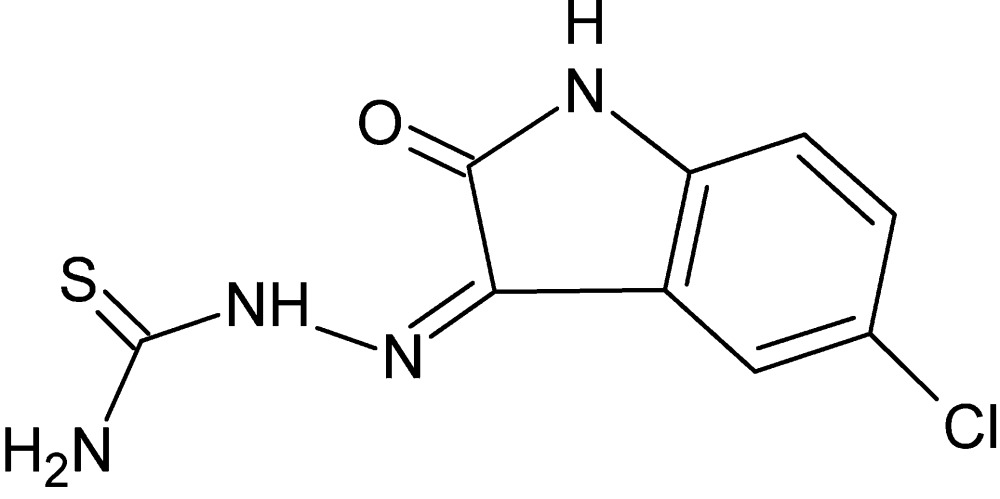


## Experimental   

### 

#### Crystal data  

C_9_H_7_ClN_4_OS
*M* *_r_* = 254.70
Monoclinic, 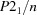

*a* = 5.260 (5) Å
*b* = 15.396 (10) Å
*c* = 13.215 (9) Å
β = 96.53 (2)°
*V* = 1063.4 (14) Å^3^
*Z* = 4
Mo *K*α radiation
μ = 0.54 mm^−1^
*T* = 173 K
1.27 × 0.38 × 0.35 mm

#### Data collection  

Bruker APEXII CCD diffractometer
Absorption correction: multi-scan (*SADABS*; Bruker, 2009 ▶) *T*_min_ = 0.639, *T*_max_ = 0.746
6939 measured reflections
2540 independent reflections
2374 reflections with *I* > 2σ(*I*)
*R*_int_ = 0.014

#### Refinement  

*R*[*F* ^2^ > 2σ(*F* ^2^)] = 0.029
*wR*(*F* ^2^) = 0.084
*S* = 0.81
2540 reflections
161 parameters
H atoms treated by a mixture of independent and constrained refinement
Δρ_max_ = 0.52 e Å^−3^
Δρ_min_ = −0.30 e Å^−3^

### 

Data collection: *APEX2* (Bruker, 2009 ▶); cell refinement: *SAINT* (Bruker, 2009 ▶); data reduction: *SAINT*; program(s) used to solve structure: *SHELXS97* (Sheldrick, 2008 ▶); program(s) used to refine structure: *SHELXL97* (Sheldrick, 2008 ▶); molecular graphics: *DIAMOND* (Brandenburg, 2006 ▶); software used to prepare material for publication: *publCIF* (Westrip, 2010 ▶).

## Supplementary Material

Crystal structure: contains datablock(s) I, New_Global_Publ_Block. DOI: 10.1107/S1600536813033369/lr2119sup1.cif

Structure factors: contains datablock(s) I. DOI: 10.1107/S1600536813033369/lr2119Isup2.hkl

Supporting information file. DOI: 10.1107/S1600536813033369/lr2119Isup3.cml

Additional supporting information:  crystallographic information; 3D view; checkCIF report

## Figures and Tables

**Table 1:** Hydrogen-bond geometry (Å, °)

*D*—H⋯*A*	*D*—H	H⋯*A*	*D*⋯*A*	*D*—H⋯*A*
N2—H21⋯O1	0.865 (18)	2.127 (18)	2.783 (2)	132.2 (15)
N4—H41⋯S1^i^	0.90 (2)	2.47 (2)	3.354 (2)	169.5 (19)
N1—H11⋯O1^ii^	0.88 (2)	1.98 (2)	2.848 (2)	169 (2)
N1—H12⋯N3	0.88 (3)	2.15 (3)	2.594 (2)	110 (2)
N1—H12⋯Cl1^iii^	0.88 (3)	2.62 (3)	3.342 (2)	139 (2)

Symmetry codes: (i) 
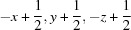
; (ii) 
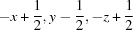
; (iii) 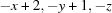.

**Fig. 1.**
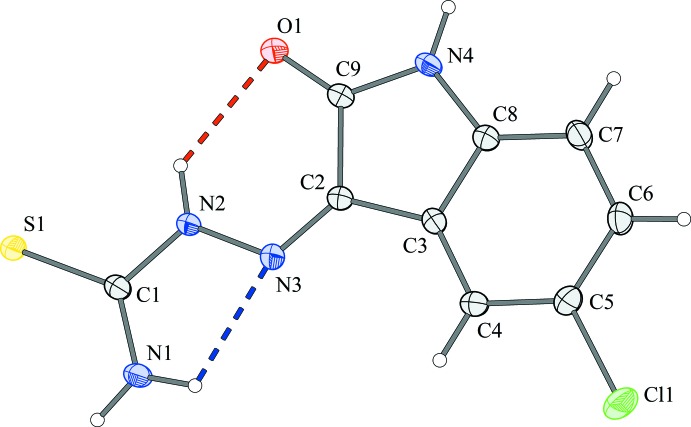
Molecular projection showing the asymmetric unit. Intramolecular hydrogen bonds are represented with dashed lines. Ellipsoid probability: 50%.

**Fig. 2.**
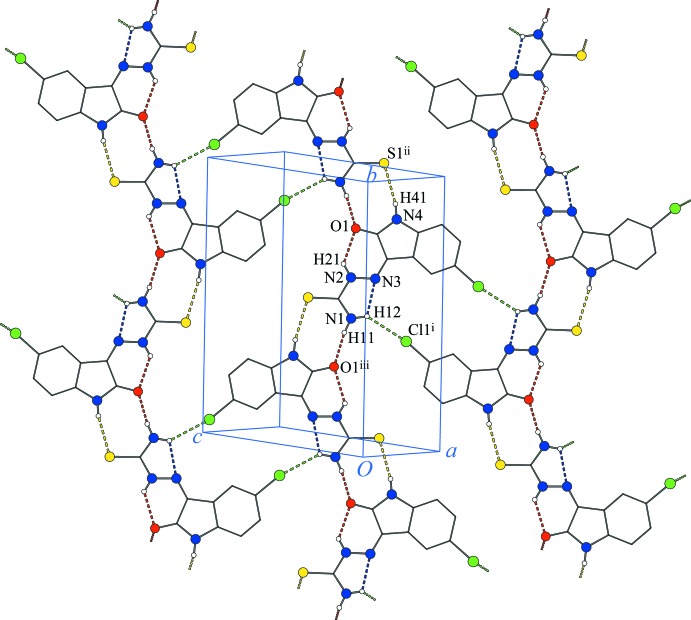
Bi-dimensional network formed through intermolecular hydrogen bonds, represented with dashed lines. Aromatic hydrogen atoms were omitted for clarity. Symmetry codes: (i) –x + 2, –y + 1, –z; (ii) –x + 1/2, y + 1/2, –z + 1/2; (iii) –x + 1/2, y - 1/2, –z + 1/2.

**Fig. 3.**
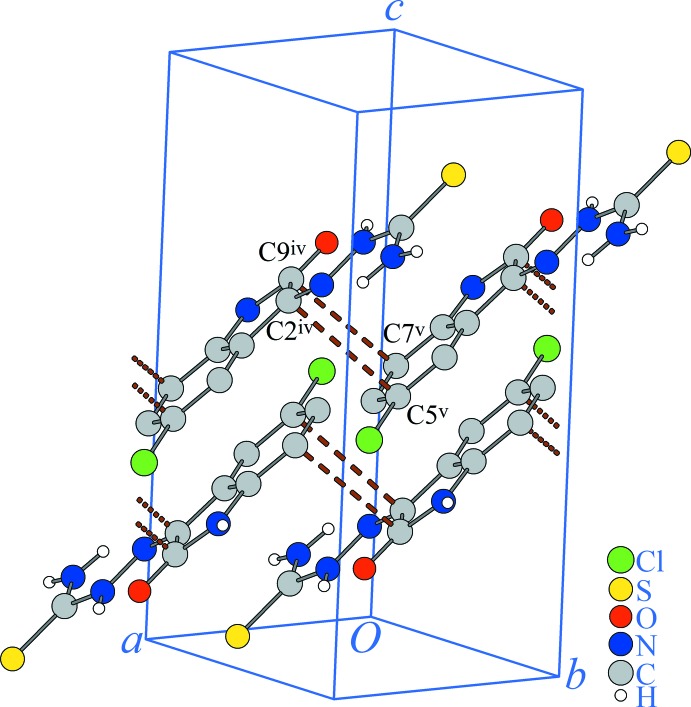
Packing of bidimensional layers through π–π stacking interactions. Aromatic hydrogen atoms were omitted for clarity. Symmetry codes: (iv) x + 1/2, –y + 3/2, z + 1/2; (v) x – 1/2, –y + 3/2, z + 1/2.

## supplementary crystallographic information

### 2.1. Synthesis and crystallization

Experimental procedures for synthesis were based on Qasem Ali *et al.* (2011). Thio­semicarbazide (0.25 g, 2.7 mmol) was mixed to 20 ml of an ethano­lic solution containing 5-chloro­isatin (0.50 g, 2.7 mmol), followed by addition of ten drops of glacial acetic acid. This mixture was maintained under reflux for 4 h, being a yellow precipitated obtained. The product was filtered off under vacuum, yielding 0.38 g (76.2%). Yellow single crystals suitable for X-ray diffraction measurements were grown in ethanol/aceto­nitrile (1:1), adding five drops of pyridine and slow evaporating at room temperature.

### 2.2. Refinement

Crystal data, data collection and structure refinement details are summarized in Table 1. H-atoms attached to aromatic C atoms were positioned with idealized geometry and were refined isotropic with *U*_eq_(H) set to 1.2 times of the *U*_eq_(C) using a riding model with C–H = 0.95 Å. H-atoms attached to N atoms were located in difference Fourier maps. Their coordinates and isotropic displacement parameters were refined.

### 3. Results and discussion

Isatin and its derivatives are known for demonstrate biological effects, such as bactericide and fungicide activities (Cerchiaro & Ferreira, 2006). This class of compounds has been characterized through X-ray crystallography: (Ali *et al.*, 2012; de Oliveira *et al.*, 2012; Bandeira *et al.*, 2013). As part of our research, in this paper we describe the synthesis (Qasem Ali *et al.*, 2011) and present the crystal structure determination of 1-(5-Chloro-2-oxoindolin-3-yl­idene)hydrazinecarbo­thio­amide.

The molecular structure of the title compound, C_9_H_7_ClN_4_OS, shows an *E* conformation about the N2–N3 bond, matching its asymmetric unit (Fig. 1). The molecule is almost planar (Bandeira *et al.*, 2013), with a r.m.s. deviation of 0.034 Å and maximum deviation from the mean plane through non-H atoms observed for the N1 atom (0.0920 (12) Å). Individually, the mean planes defined through non-H atoms of the thio­semicarbazone fragment (S1/C1/N1–N3) and the chloro substituted aromatic ring (C3–C8/Cl1) reveals a dihedral angle of 4.75 (8)°, with maximum deviations of 0.0058 (11) Å and 0.0043 (11) Å, respectively (de Oliveira *et al.*, 2012).

Intra-molecular N1–H12···N3 (2.15 (3) Å) and N2–H21···O1 (2.127 (18) Å) hydrogen bonds are observed, forming *S*(5) and *S*(6) ring motifs, respectively. Dimeric species are formed through inter­molecular N3–H12···Cl1^i^ hydrogen bonds with a distance of 2.62 (3) Å (symmetry code: (i) –*x* + 2, –*y* + 1, –*z*) and molecular units being related by crystallographic centers of symmetry. Shorter inter­molecular hydrogen bonding with distances of 2.47 (2) Å (N4–H41···S1^ii^) and 1.98 (2) Å (N1–H11···O1^iii^) occur in a *R*_4_^4^(8) ring fashion, connecting molecules into a bi-dimensional net parallel to the [103] plane (Fig. 2, symmetry codes: (ii) –*x* + 1/2, *y* + 1/2, –*z* + 1/2; (iii) –*x* + 1/2, *y* - 1/2, –*z* + 1/2). Additional weak π–π stacking inter­actions are verified between adjacent bi-dimensional layers along the *a* axis, with C2^iv^···C5^v^ and C9^iv^···C7^v^ distances of 3.3209 (26) Å and 3.3973 (28) Å, respectively (Fig. 3, symmetry codes: (iv) *x* + 1/2, –*y* + 3/2, *z* + 1/2; (v) *x* – 1/2, –*y* + 3/2, *z* + 1/2). All these bonding features are similar to that described by Bandeira *et al.* (2013), despite of the different crystal symmetry verified previously.

### Crystal data

**Table:**

C_9_H_7_ClN_4_OS	*Z* = 4
*M**_r_* = 254.70	*F*(000) = 520
Monoclinic, *P*2_1_/*n*	*D*_x_ = 1.591 Mg m^−^^3^
Hall symbol: -P 2yn	Mo *K*α radiation, λ = 0.71073 Å
*a* = 5.260 (5) Å	θ = 2.0–28.3°
*b* = 15.396 (10) Å	µ = 0.54 mm^−^^1^
*c* = 13.215 (9) Å	*T* = 173 K
β = 96.53 (2)°	Block, yellow
*V* = 1063.4 (14) Å^3^	1.27 × 0.38 × 0.35 mm

### Data collection

**Table:**

Bruker APEXII CCD diffractometer	2540 independent reflections
Radiation source: fine-focus sealed tube	2374 reflections with *I* > 2σ(*I*)
Graphite monochromator	*R*_int_ = 0.014
φ and ω scans	θ_max_ = 28.3°, θ_min_ = 2.0°
Absorption correction: multi-scan (*SADABS*; Bruker, 2009)	*h* = −6→7
*T*_min_ = 0.639, *T*_max_ = 0.746	*k* = −20→18
6939 measured reflections	*l* = −17→17

### Refinement

**Table:**

Refinement on *F*^2^	Primary atom site location: structure-invariant direct methods
Least-squares matrix: full	Secondary atom site location: difference Fourier map
*R*[*F*^2^ > 2σ(*F*^2^)] = 0.029	Hydrogen site location: inferred from neighbouring sites
*wR*(*F*^2^) = 0.084	H atoms treated by a mixture of independent and constrained refinement
*S* = 0.81	*w* = 1/[σ^2^(*F*_o_^2^) + (0.0594*P*)^2^ + 1.1793*P*] where *P* = (*F*_o_^2^ + 2*F*_c_^2^)/3
2540 reflections	(Δ/σ)_max_ = 0.001
161 parameters	Δρ_max_ = 0.52 e Å^−^^3^
0 restraints	Δρ_min_ = −0.30 e Å^−^^3^

### Special details

**Table:**

Geometry. All e.s.d.'s (except the e.s.d. in the dihedral angle between two l.s. planes) are estimated using the full covariance matrix. The cell e.s.d.'s are taken into account individually in the estimation of e.s.d.'s in distances, angles and torsion angles; correlations between e.s.d.'s in cell parameters are only used when they are defined by crystal symmetry. An approximate (isotropic) treatment of cell e.s.d.'s is used for estimating e.s.d.'s involving l.s. planes.
Refinement. Refinement of *F*^2^ against ALL reflections. The weighted *R*-factor *wR* and goodness of fit *S* are based on *F*^2^, conventional *R*-factors *R* are based on *F*, with *F* set to zero for negative *F*^2^. The threshold expression of *F*^2^ > σ(*F*^2^) is used only for calculating *R*-factors(gt) *etc*. and is not relevant to the choice of reflections for refinement. *R*-factors based on *F*^2^ are statistically about twice as large as those based on *F*, and *R*- factors based on ALL data will be even larger.

### Fractional atomic coordinates and isotropic or equivalent isotropic displacement parameters (Å^2^)

**Table:**

	*x*	*y*	*z*	*U*_iso_*/*U*_eq_	
Cl1	1.30216 (7)	0.60089 (2)	−0.07671 (3)	0.02850 (11)	
S1	0.02044 (6)	0.54702 (2)	0.36977 (3)	0.01934 (10)	
O1	0.35742 (18)	0.80256 (6)	0.24115 (7)	0.0187 (2)	
N1	0.3377 (3)	0.47463 (8)	0.24996 (11)	0.0285 (3)	
N2	0.3459 (2)	0.62260 (7)	0.25988 (8)	0.0162 (2)	
N3	0.5262 (2)	0.62044 (7)	0.19443 (8)	0.0159 (2)	
N4	0.6662 (2)	0.83859 (7)	0.13477 (8)	0.0166 (2)	
C1	0.2444 (2)	0.54529 (8)	0.28857 (10)	0.0163 (2)	
C2	0.6101 (2)	0.69327 (8)	0.16351 (9)	0.0148 (2)	
C3	0.8029 (2)	0.70274 (8)	0.09346 (9)	0.0152 (2)	
C4	0.9460 (2)	0.64250 (8)	0.04630 (10)	0.0173 (2)	
H4	0.9271	0.5819	0.0565	0.021*	
C5	1.1187 (2)	0.67488 (9)	−0.01663 (10)	0.0189 (3)	
C6	1.1518 (2)	0.76349 (9)	−0.03214 (10)	0.0192 (3)	
H6	1.2728	0.7829	−0.0753	0.023*	
C8	0.8326 (2)	0.79228 (8)	0.07785 (9)	0.0152 (2)	
C9	0.5256 (2)	0.78350 (8)	0.18693 (10)	0.0156 (2)	
C7	1.0070 (3)	0.82389 (9)	0.01575 (10)	0.0180 (3)	
H7	1.0274	0.8845	0.0061	0.022*	
H21	0.291 (3)	0.6714 (12)	0.2814 (13)	0.017 (4)*	
H41	0.637 (4)	0.8958 (15)	0.1304 (16)	0.035 (5)*	
H11	0.276 (4)	0.4232 (15)	0.2616 (16)	0.033 (5)*	
H12	0.459 (5)	0.4835 (18)	0.210 (2)	0.054 (7)*	

### Atomic displacement parameters (Å^2^)

**Table:**

	*U*^11^	*U*^22^	*U*^33^	*U*^12^	*U*^13^	*U*^23^
Cl1	0.02824 (19)	0.0271 (2)	0.0332 (2)	0.00156 (13)	0.01668 (14)	−0.00782 (13)
S1	0.02276 (18)	0.01321 (17)	0.02450 (18)	−0.00194 (11)	0.01337 (13)	−0.00236 (11)
O1	0.0228 (5)	0.0133 (4)	0.0215 (5)	0.0022 (3)	0.0088 (4)	0.0002 (3)
N1	0.0363 (7)	0.0112 (5)	0.0437 (8)	−0.0024 (5)	0.0288 (6)	−0.0028 (5)
N2	0.0209 (5)	0.0099 (5)	0.0195 (5)	0.0005 (4)	0.0093 (4)	0.0002 (4)
N3	0.0180 (5)	0.0136 (5)	0.0171 (5)	0.0003 (4)	0.0067 (4)	0.0007 (4)
N4	0.0199 (5)	0.0094 (5)	0.0216 (5)	0.0005 (4)	0.0066 (4)	0.0016 (4)
C1	0.0183 (6)	0.0117 (6)	0.0196 (6)	−0.0004 (4)	0.0051 (5)	0.0006 (4)
C2	0.0169 (5)	0.0117 (5)	0.0163 (5)	0.0010 (4)	0.0040 (4)	0.0002 (4)
C3	0.0168 (5)	0.0127 (6)	0.0165 (5)	−0.0006 (4)	0.0044 (4)	0.0016 (4)
C4	0.0191 (6)	0.0132 (6)	0.0202 (6)	0.0003 (4)	0.0050 (5)	−0.0010 (4)
C5	0.0184 (6)	0.0194 (6)	0.0198 (6)	0.0011 (5)	0.0061 (5)	−0.0014 (5)
C6	0.0179 (6)	0.0216 (7)	0.0191 (6)	−0.0028 (5)	0.0059 (5)	0.0018 (5)
C8	0.0164 (6)	0.0132 (6)	0.0162 (6)	0.0006 (4)	0.0024 (4)	0.0007 (4)
C9	0.0190 (6)	0.0111 (5)	0.0170 (6)	0.0004 (4)	0.0035 (4)	0.0008 (4)
C7	0.0199 (6)	0.0157 (6)	0.0187 (6)	−0.0014 (5)	0.0037 (5)	0.0036 (4)

### Geometric parameters (Å, º)

**Table:**

Cl1—C5	1.7417 (16)	N4—H41	0.90 (2)
S1—C1	1.6816 (17)	C2—C3	1.4561 (19)
O1—C9	1.2354 (18)	C2—C9	1.5015 (19)
N1—C1	1.3196 (18)	C3—C4	1.3863 (18)
N1—H11	0.88 (2)	C3—C8	1.4052 (19)
N1—H12	0.88 (3)	C4—C5	1.3919 (19)
N2—N3	1.3548 (17)	C4—H4	0.9500
N2—C1	1.3753 (17)	C5—C6	1.393 (2)
N2—H21	0.865 (18)	C6—C7	1.398 (2)
N3—C2	1.2886 (18)	C6—H6	0.9500
N4—C9	1.3629 (17)	C8—C7	1.3869 (19)
N4—C8	1.4108 (17)	C7—H7	0.9500
			
C1—N1—H11	121.0 (14)	C3—C4—C5	116.98 (13)
C1—N1—H12	115.4 (18)	C3—C4—H4	121.5
H11—N1—H12	124 (2)	C5—C4—H4	121.5
N3—N2—C1	118.47 (11)	C4—C5—C6	122.65 (12)
N3—N2—H21	121.0 (12)	C4—C5—Cl1	118.11 (11)
C1—N2—H21	120.5 (12)	C6—C5—Cl1	119.23 (11)
C2—N3—N2	118.11 (11)	C5—C6—C7	120.06 (12)
C9—N4—C8	111.12 (11)	C5—C6—H6	120.0
C9—N4—H41	123.0 (14)	C7—C6—H6	120.0
C8—N4—H41	125.2 (14)	C7—C8—C3	121.54 (12)
N1—C1—N2	115.72 (13)	C7—C8—N4	129.09 (12)
N1—C1—S1	125.31 (10)	C3—C8—N4	109.36 (11)
N2—C1—S1	118.97 (10)	O1—C9—N4	127.66 (12)
N3—C2—C3	125.26 (11)	O1—C9—C2	126.00 (11)
N3—C2—C9	128.28 (12)	N4—C9—C2	106.33 (11)
C3—C2—C9	106.41 (10)	C8—C7—C6	117.75 (13)
C4—C3—C8	121.02 (12)	C8—C7—H7	121.1
C4—C3—C2	132.21 (12)	C6—C7—H7	121.1
C8—C3—C2	106.77 (11)		
			
C1—N2—N3—C2	176.25 (12)	C4—C3—C8—C7	−0.62 (19)
N3—N2—C1—N1	1.08 (19)	C2—C3—C8—C7	178.86 (11)
N3—N2—C1—S1	180.00 (9)	C4—C3—C8—N4	−179.71 (11)
N2—N3—C2—C3	−179.99 (11)	C2—C3—C8—N4	−0.23 (14)
N2—N3—C2—C9	−3.0 (2)	C9—N4—C8—C7	−179.50 (12)
N3—C2—C3—C4	−2.3 (2)	C9—N4—C8—C3	−0.49 (15)
C9—C2—C3—C4	−179.81 (13)	C8—N4—C9—O1	−177.55 (12)
N3—C2—C3—C8	178.32 (12)	C8—N4—C9—C2	0.97 (14)
C9—C2—C3—C8	0.79 (14)	N3—C2—C9—O1	0.0 (2)
C8—C3—C4—C5	−0.01 (19)	C3—C2—C9—O1	177.47 (12)
C2—C3—C4—C5	−179.33 (13)	N3—C2—C9—N4	−178.51 (13)
C3—C4—C5—C6	0.6 (2)	C3—C2—C9—N4	−1.08 (13)
C3—C4—C5—Cl1	179.81 (9)	C3—C8—C7—C6	0.66 (19)
C4—C5—C6—C7	−0.5 (2)	N4—C8—C7—C6	179.56 (12)
Cl1—C5—C6—C7	−179.75 (10)	C5—C6—C7—C8	−0.11 (19)

### Hydrogen-bond geometry (Å, º)

**Table:**

*D*—H···*A*	*D*—H	H···*A*	*D*···*A*	*D*—H···*A*
N2—H21···O1	0.865 (18)	2.127 (18)	2.783 (2)	132.2 (15)
N4—H41···S1^i^	0.90 (2)	2.47 (2)	3.354 (2)	169.5 (19)
N1—H11···O1^ii^	0.88 (2)	1.98 (2)	2.848 (2)	169 (2)
N1—H12···N3	0.88 (3)	2.15 (3)	2.594 (2)	110 (2)
N1—H12···Cl1^iii^	0.88 (3)	2.62 (3)	3.342 (2)	139 (2)

Symmetry codes: (i) −*x*+1/2, *y*+1/2, −*z*+1/2; (ii) −*x*+1/2, *y*−1/2, −*z*+1/2; (iii) −*x*+2, −*y*+1, −*z*.
